# A Flexible and Stretchable MXene/Waterborne Polyurethane Composite-Coated Fiber Strain Sensor for Wearable Motion and Healthcare Monitoring

**DOI:** 10.3390/s24010271

**Published:** 2024-01-02

**Authors:** Junming Cao, Yuanqing Jiang, Xiaoming Li, Xueguang Yuan, Jinnan Zhang, Qi He, Fei Ye, Geng Luo, Shaohua Guo, Yangan Zhang, Qi Wang

**Affiliations:** 1 State Key Laboratory of Information Photonics and Optical Communications, Beijing University of Posts and Telecommunications, Beijing 100876, China; 2 School of Electronic Engineering, Beijing University of Posts and Telecommunications, Beijing 100876, China; 3 No. 208 Research Institute of China Ordnance Industries, Beijing 102202, China; 15810098180@163.com (Y.J.);

**Keywords:** MXene, polyurethane, fiber, dip coating, strain sensor, motion detection, body posture, healthcare

## Abstract

Fiber-based flexible sensors have promising application potential in human motion and healthcare monitoring, owing to their merits of being lightweight, flexible, and easy to process. Now, high-performance elastic fiber-based strain sensors with high sensitivity, a large working range, and excellent durability are in great demand. Herein, we have easily and quickly prepared a highly sensitive and durable fiber-based strain sensor by dip coating a highly stretchable polyurethane (PU) elastic fiber in an MXene/waterborne polyurethane (WPU) dispersion solution. Benefiting from the electrostatic repulsion force between the negatively charged WPU and MXene sheets in the mixed solution, very homogeneous and stable MXene/WPU dispersion was successfully obtained, and the interconnected conducting networks were correspondingly formed in a coated MXene/WPU shell layer, which makes the as-prepared strain sensor exhibit a gauge factor of over 960, a large sensing range of over 90%, and a detection limit as low as 0.5% strain. As elastic fiber and mixed solution have the same polymer constitute, and tight bonding of the MXene/WPU conductive composite on PU fibers was achieved, enabling the as-prepared strain sensor to endure over 2500 stretching–releasing cycles and thus show good durability. Full-scale human motion detection was also performed by the strain sensor, and a body posture monitoring, analysis, and correction prototype system were developed via embedding the fiber-based strain sensors into sweaters, strongly indicating great application prospects in exercise, sports, and healthcare.

## 1. Introduction

Flexible strain sensors have received great attention due to their high efficiency in converting mechanical deformations in the human body into electrical signals, enabling their broad applications in human motion detection, sports, real-time healthcare monitoring, etc. [1,2,3,4,5,6,7]. Among all types of stretchable substrates needed for flexible sensors, fibers are one of the competitive choices because of their virtues of being lightweight and flexible and their embeddability, which allows for their seamless connection with fabrics and cloth [8,9,10,11,12,13,14]. Now, the simple, mass, and reproducible fabrication of fiber-based flexible strain sensors with excellent performance is required. For such intention, several methods, such as wet spinning [15,16,17] and dip coating [18,19,20], have been reported. For example, Huang et al. developed a coaxial fiber-based strain sensor with high sensitivity and a wide detection range by a continuous, facile, and scalable wet spinning approach [21]. However, the wet spinning process generally includes the formation of fine stream by pressing, as well as the production of fibers by coagulation in the solution, which is often completed by the specific equipment or apparatus. Dip coating is another widely adopted facile approach, which normally deposits a conductive layer on a fiber surface, thus holding the advantage of high efficiency and low equipment requirements over wet spinning. For instance, in our previous work, a fiber-based strain sensor was fabricated by simply hydrothermally reducing the dip-coated graphene oxide on elastic fibers of yarns, and human motion monitoring, sports monitoring, healthcare monitoring, and gesture recognition have been successfully demonstrated by them [22,23,24,25]. Nevertheless, we find that dip-coated solid conductive materials have relatively poor adhesion to the fiber, and a mechanical property mismatch exists between them, which makes the solid coating layer difficult to endure daily rubbing or numerous stretching–releasing cycles of the fiber sensor in actual usage scenarios. Considering the robustness, adhesiveness, controllable electrical properties, and mechanical properties of the dip coating layer, conductive polymer composites (CPCs), which consist of conductive filler and a polymer matrix, have become an ideal substitute [26,27,28,29,30,31,32].

Recently, a new large family of two-dimensional (2D) transition metal carbides or nitrides, called MXene, has become a very hot conductive filler for CPCs and has gradually shown superiority to the counterparts already used by strain sensors due to its metallic conductivity and high specific surface area [33,34,35,36,37,38]. Moreover, abundant polar functional groups generated during the liquid phase etching process endow MXene with excellent hydrophilicity, enabling and facilitating their solution-based employment approaches [39,40,41]. In addition, with the rise of environmental awareness and the use of non-pollution and low-toxicity polymers has become another trend for CPCs [42,43,44,45]. Waterborne polyurethane (WPU), a new type of PU system that uses water instead of organic solvents as a dispersion medium, has abundant hydrophilic functional groups, so it can form a strong bond to various substrates through hydrogen bonding, showing excellent film-forming properties and processability and thus attracting much attention [46,47,48]. However, MXene nanoflake or nanosheet dispersions are susceptible to stacking in a WPU solution, which makes it difficult to construct uniform and stable CPC dispersions. Fortunately, such a dilemma can be effectively removed or suppressed by introducing an electrostatic repulsion mechanism into MXene/WPU composites [49,50,51], i.e., a negatively charged WPU was chosen to mix with negatively charged MXene. At the same time, if a PU elastic fiber was accepted for dip coating, fiber and CPC dispersion would have the same PU polymer constituent, and the mechanical mismatch of Young’s modulus between the fiber and CPC would be significantly reduced; thus, very strong bonding of the CPC and PU fiber can be expected.

Herein, homogeneous MXene/WPU composite dispersion was prepared by blending a negatively charged WPU solution and an MXene nanosheet solution, which was then dip coated on a PU elastic fiber matrix to prepare an elastic fiber-based strain sensor. The as-prepared fiber strain sensor exhibited attractive sensing performance, including high sensitivity with a gauge factor of over 960, a broad sensing range of over 90%, and a low detection limit of 0.5%; thus, full-scale human motion detection from vigorous joint movement to subtle expression change and wrist pulse was realized. Moreover, benefiting from the tight bonding between the MXene/WPU composite coating layer and PU fiber, the fiber strain sensors exhibited good washing and peeling resistant capability, as well as good durability of over 2500 stretching–releasing cycles, which is robust enough to endure daily human activities. Finally, a smart data glove for finger bending angle and hand gesture monitoring and a prototype body posture monitoring and correction system were developed by embedding the fiber-based strain sensors into common fabrics, demonstrating the great potential of as-fabricated wearable, comfortable, non-intrusive sensors in exercise, sports, gesture recognition, and healthcare applications.

## 2. Materials and Methods

### 2.1. Materials

The Ti_3_AlC_2_ MAX phase (400 mesh) was purchased from Jilin 11 Technology Co., Ltd. (Jilin, China). Lithium fluoride (LiF, 99.9% purity) and hydrochloric acid (HCl, 37% concentration) were purchased from Aladdin Reagent Co., Ltd. (Shanghai, China). WPU (35 wt%) with good adhesion (300 mpa·S in viscosity) was purchased from Shenzhen Jitian Chemical Co., Ltd. (Shenzhen, China). PU elastic fibers (~500 µm in diameter) were purchased from Jiang Su Geruite Textile Co., Ltd. (Danyang, China).

### 2.2. Synthesis of MXene Nanosheet Suspension

A total of 40 mL of 9 M HCl acid and 2 g LiF powder were slowly added into a Teflon container. Magnetic stirring was employed to obtain sufficient dissolution and mixing. Then, 3 g of Ti_3_AlC_2_ MAX precursor powder was slowly added into the above-mixed solution for etching, and the whole etching procedure was kept for 24 h at 35 °C. After full reaction and etching, the obtained suspension was centrifugally separated (4500 rpm, 4 min) and washed with deionized water several times until the pH value reached about 6. After that, the obtained black sediment was diluted with an appropriate amount of deionized water and delaminated under sonication. Then, the mixture was further centrifugally separated (3000 rpm, 30 min), and the dark green middle solution was collected as the MXene nanosheet suspension. The typical fabrication steps of Ti_3_AlC_X_ MXene from the Ti_3_AlC_2_ MAX precursor were shown by SEM images and XRD patterns in Figure S1.

### 2.3. Preparation of the MXene/WPU Composite-Coated Fiber Sensor

**Figure 1:**
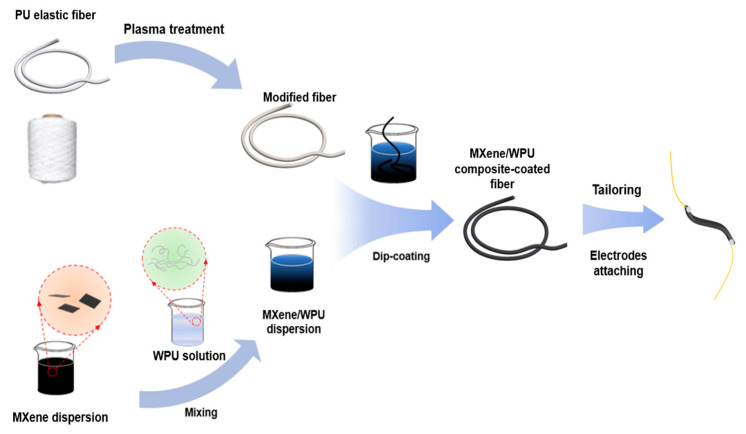
Schematics of the fabrication flows of the MXene/WPU composite-coated fiber sensor.

The fabrication process of the MXene/WPU composite-coated fiber sensor is shown in Figure 1. The MXene/WPU dispersion solution with various MXene/WPU mass ratios (1:1, 1:3, 1:5, and 1:7) was obtained by adding the negatively charged WPU (PU, 35 wt%) solution into a specific amount of Ti_3_C_2_Tx MXene suspension. To become fully mixed, the MXene/WPU solution was then magnetic stirred for 1 h at room temperature.

PU elastic fibers were washed thoroughly with ethanol and DI water for 5 min, respectively, and then transferred into an oven to dry at 50 °C for 1 h. Next, the elastic fibers were treated by glow discharge air plasma for 10 min to increase the surface roughness and generate polar groups [52]. Subsequently, the elastic fibers were dipped into an MXene/WPU dispersion solution for a 15 min ultrasonic treatment and transferred into the oven to dry at 50 °C for 1 h again. After drying, a CPC layer was successfully coated on the surface of elastic fibers. A series of MXene/WPU composite-coated elastic fibers were fabricated and cut into short sections with equal lengths of 4 cm. With the assistance of conductive silver glue, both ends of the tailored fibers were connected with 5 cm copper wire electrodes, finally forming the fiber-based strain sensor.

### 2.4. Characterization

Zeta potentials of Ti_3_C_2_T_x_ suspensions and WPU were determined by a nanoparticle size and zeta potential analyzer (Zetasizer Nano ZS90, Malvern, UK). The morphologies of the MXene/WPU composite coating layer were observed using a field emission scanning electron microscope (Merlin, Carl Zeiss, Jena, Germany). The strain sensing performance of the MXene/WPU composite-coated fiber strain sensor was measured by a simple coupling system that consists of a universal testing machine (AG-XplusHS 1 kN, Shimadzu, Japan), a source meter (B2902, Keithley, Cleveland, OH, USA), and a computer. A cyclic test of the fiber sensor was conducted by applying circulative strains through an algorithm-controlled stepper motor.

## 3. Results and Discussion

### 3.1. Morphologies of MXene/WPU Composite-Coated Fiber

**Figure 2:**
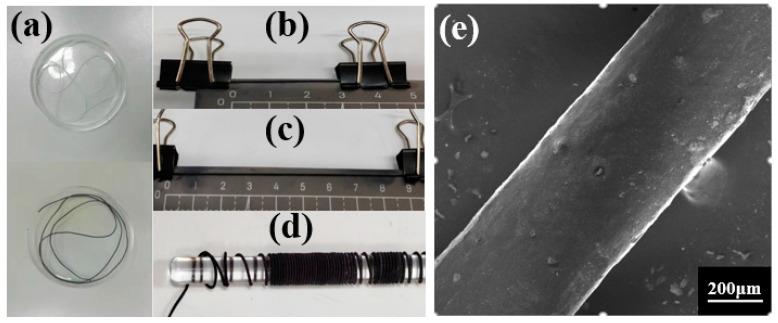
(**a**) Photographic comparison of untreated fiber and MXene/WPU composite-coated fiber; photographs of MXene/WPU composite-coated fiber (**b**) without any strain, (**c**) under 300% strain, and (**d**) wound on a perspex bar. (**e**) SEM image of MXene/WPU composite-coated fiber.

After a one-time dip coating process, MXene/WPU composite conductive layers were successfully coated on the surface of PU elastic fibers, accompanied by an obvious change in fiber color from white to black, as shown in Figure 2a. As presented in Figure 2b–d, the coated fibers endured a large strain of 300% and were also tightly wound on a thick perspex bar, showing good stretchability and favorable flexibility, respectively. Without any conductive treatment to the sample, such as gold sputtering or spraying carbon powder, a magnified SEM image of the coated fiber is obtained and shown in Figure 2e, which indicates that the MXene/WPU CPC layer has an excellent coating layer. Moreover, the coated fiber held a uniform cylindrical structure and smooth surface, which is beneficial to the future weaving or application of the coated fibers.

**Figure 3:**
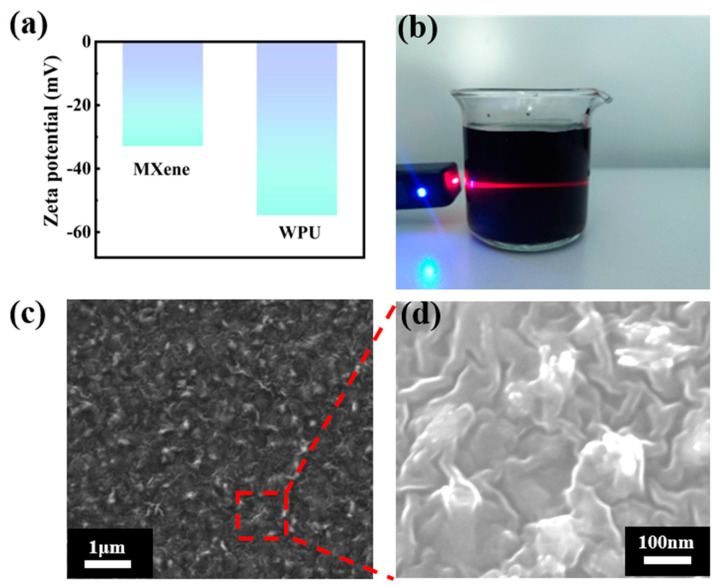
(**a**) Zeta potentials of Ti_3_C_2_T_x_ and WPU solutions. (**b**) Tyndall effect observed from MXene/WPU dispersion. (**c**,**d**) SEM images of the MXene/WPU composite layer coated on PU fiber.

As indicated by the measured zeta potentials (Figure 3a), both MXene nanosheets and WPU molecules have adequate negative charges, which guarantees the formation of homogeneous MXene/WPU dispersion under the electrostatic repulsion between them. As shown in Figure 3b, a very clear Tyndall effect was observed with the naked eye, providing powerful evidence of homogeneous and stable dispersion of MXene nanosheets in a WPU solution [53]. Furthermore, as shown in Figure 3c,d, magnified SEM images of MXene/WPU composites show the uniform distribution of MXene nanosheets in the WPU polymer, which validates the homogeneous dispersion again.

### 3.2. Strain Sensing Performance of MXene/WPU Composite-Coated Fiber

**Figure 4:**
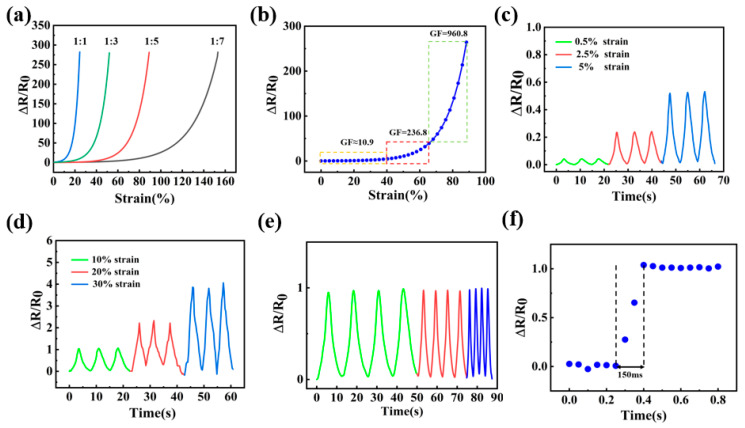
(**a**) Resistance variant curves of MXene/WPU composite-coated fibers with four MXene/WPU mass ratios under tensile strain and (**b**) the magnified curve for the MXene/WPU mass ratio of 1:5. Cyclic sensing behaviors of the sensor under (**c**) tiny strain, (**d**) large strain, and (**e**) fixed 10% strain with variable frequencies. (**f**) Response time of the sensor to quasi-transient step strain (10% strain).

Firstly, resistance variations of MXene/WPU composite-coated fibers with different MXene content were systematically investigated under the continuously increased strain, as shown in Figure 4a. All of the fiber samples displayed a resistance ramping trend along with the strain increment, which was ascribed to the destruction of the conductive network upon external tension. In addition, higher MXene content in the composite will induce a higher gauge factor (GF) of the fiber and a significant reduction in the stretchable range or the affordable strain. According to the overall strain sensing performance, MXene/WPU composite-coated fiber with MXene content of 16.7% (i.e., MXene/WPU mass ratio = 1:5) was selected as the sole fiber sensor sample for the following tests. Figure 4b shows the resistance variation curve of the sensor sample under strain ranging from 0 to 90%, which can be further divided into three sections: 0–40% strain (GF ≈ 10.9), 40–65% strain (GF ≈ 236.8), and 65–90% strain (GF ≈ 960.8). Quickly enhanced sensitivities were ascribed and found that much more conductive networks in the coated MXene/WPU composite were destroyed when great strain was exerted. Cyclic strain sensing behaviors of the sensor under tiny strain (0.5–5% strain) and large strain (10–30% strain) are comprehensively investigated and shown in Figure 4c,d, from which it can be clearly seen that relative sensor resistance ramps up with increasing strain, and the resistance of the sensor almost completely returns to the initial value once releasing the strain, exhibiting outstanding reproducibility and stability. Furthermore, the electrical response of the composite-coated fibers to a fixed strain of 10% under the frequency range of 0.08–0.32 Hz was investigated and shown in Figure 4e, from which the resistance variation of the sensor was negligibly affected by applied frequencies, thus indicating an excellent dynamic reliability. As shown in Figure 4f, the sensor shows a rapid response time of 150 ms when exerting a quasi-transient step strain of 10%, making it feasible to be used in fields such as human motion monitoring, which generally requires timely feedback. 

### 3.3. Robustness and Durability of MXene/WPU Composite-Coated Fiber

**Figure 5:**
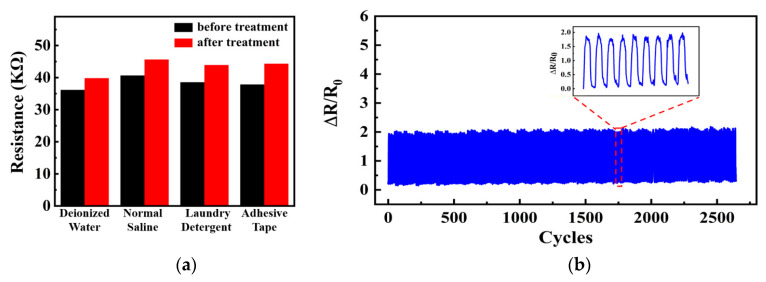
Resistance changes in the sensor under (**a**) various treatments and (**b**) >2500 repeated stretching–releasing cycles.

Three trial groups of the same MXene/PU composite-coated elastic fiber sensor samples were immersed in DI water, a saline solution, and a laundry detergent solution, respectively, and all were ultrasonicated for 1 h, simulating the exposure of the sensor sample to rain washing, sweat soaking, and washing in daily life. In addition, a piece of transparent adhesive tape was also tightly attached to the CPC layer and then suddenly peeled off, artificially creating the mechanical wear of the sensor sample from rubbing. As shown in Figure 5a, the average resistance value of the sensor sample only showed a slight change of <10% against the harsh treatments, showing adequate robustness of the sensor samples. Furthermore, the sensor sample was further evaluated by a cyclic stretching–releasing test (>2500 cycles) under 20% fixed strain, as shown in Figure 5b, and a stable sensing response was clearly recorded, further showing good durability of the sensor samples. 

### 3.4. Strain Sensing Mechanism Analysis

**Figure 6:**
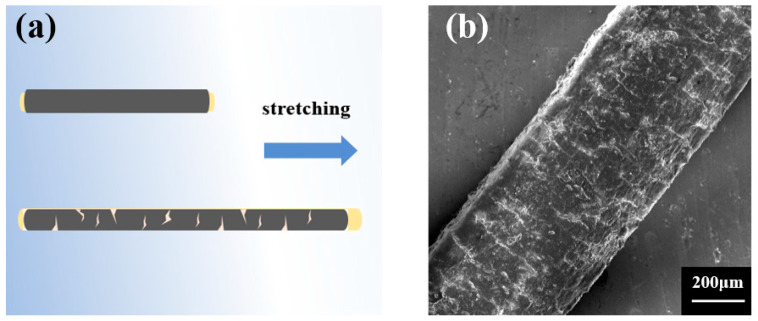
(**a**) Schematic illustrations showing the sensing mechanism of the MXene/WPU composite-coated fiber (**b**) SEM image of the MXene/WPU composite-coated fiber under stretching.

The sensing mechanism of the fiber strain sensor was revealed by investigating the structural change in the MXene/WPU CPC layer during the stretching–releasing cycle. As illustrated in Figure 6a, the CPC shell layer was extended under sensor stretching, and obvious microcracks that lengthen the transmission pathway of the electrons and thus increase the resistance of the sensor will be generated in the CPC layer, which were further verified by the SEM image of the stretched fiber sensor in Figure 6b. Upon releasing the sensor, the microcracks will close again, which makes the previous prolonged conductive pathways recover to the original state again and thus induces resistance decrease [54,55]. Obviously, it is very reasonable to believe that the obtained strain-sensing performance is ascribed to those microcracks.

### 3.5. Full-Scale Human Motion Detection

**Figure 7:**
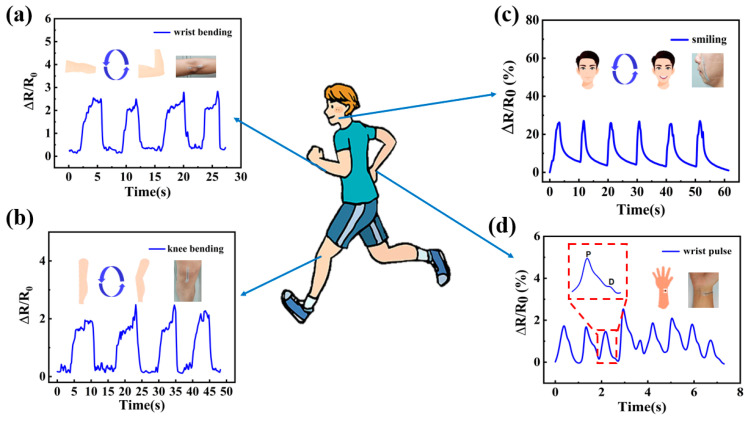
Resistance variation curves of the fiber strain sensor when measuring (**a**) wrist bending, (**b**) knee bending, (**c**) smiling, and (**d**) wrist pulse.

Favorable sensing performance obtained by the fiber strain sensor, including very high sensitivity, wide working range, excellent robustness, and long-term durability, strongly enables them to be attractive candidates for wearable electronic applications. For the applicable validation, the sensor samples were directly mounted on the corresponding body parts of one young guy to continuously record the electrical signal waveform when he exerts full-scale human motions, including intense human activities, like joint movements, and subtle human activities, like expressions and wrist pulses. As shown in Figure 7a,b, a reproducible signal waveform was clearly recorded when the volunteer performed the bending and releasing movement of his elbow joint, and a knee movement waveform was observed when walking or running was also repeatedly exhibited, both demonstrating the ability of the sensor samples to detect high-intensity motions. As shown in Figure 7c,d, thanks to the high sensitivity, the sensor mounted on the face successfully tracked the subtle facial expression changes from silence to smiling, and the resistance of the strain sensor also varied regularly with the wrist pulse wave; additionally, the percussion wave (P-wave) and the diastolic wave (D-wave) can be easily distinguished in one complete pulse wave.

### 3.6. Applications for Wearable Gesture and Healthcare Monitoring

**Figure 8:**
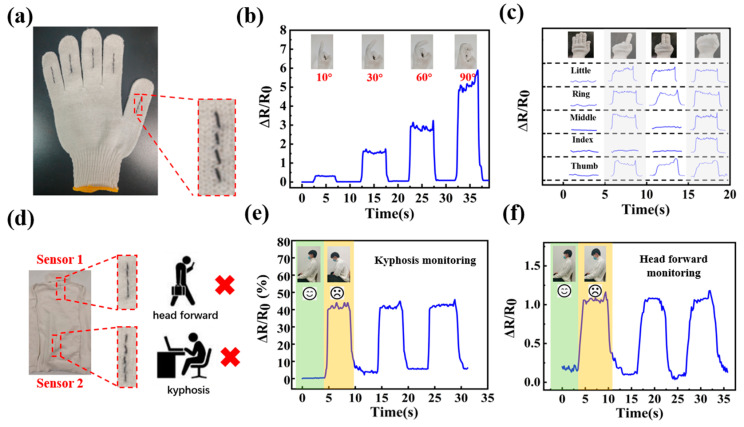
(**a**) A smart data glove woven with five fiber strain sensors onto the joints of the five fingers. The electrical signal output of the fiber strain sensors on the data glove was used for monitoring different (**b**) bending angles of each finger and (**c**) gestures performed by five fingers. (**d**) A prototype wearable system based on smart clothing woven with two fiber strain sensors was developed. The electrical signal output of the fiber strain sensors was utilized for monitoring (**e**) kyphsis and (**f**) head forward.

Benefiting from the good embeddability and processability brought by the fiber as a typical 1D structure, an elastic fiber-based strain sensor can be woven into various fabrics or cloth so as to achieve more advanced wearable sensing and monitoring physiological conditions and motion activities. As shown in Figure 8a, a flexible data glove was fabricated by sewing five discrete fiber strain sensors onto the articulation positions of one cotton string glove, and every sensor can accurately monitor the bending angle of the corresponding finger joint. Taking the index finger as an example, as shown in Figure 8b, different angular positions (10°, 30°, 60°, and 90°) of the bending finger can be distinguished by the sewed fiber strain sensor. Furthermore, as illustrated in Figure 8c, simple gestures made by the data glove can be readily recognized by comprehensively evaluating the intensity and patterns of the detected five-channel signals. Healthcare monitoring application of the fiber strain sensors was also investigated. As is well known already, poor sitting postures, such as head forward and kyphosis, will lead to many musculoskeletal disorders; therefore, research on posture monitoring and correcting is of great importance and has been a hot focus. In this study, using the fiber strain sensors as the core sensing components, a simple prototype system has been successfully developed. In detail, as shown in Figure 8d, smart clothing was prepared by sewing fiber strain sensors onto different parts (marked red) of the back region of a turtleneck tight sweater, in which sensor 1 and sensor 2 are used to monitor neck movement and back bending, respectively. The sensitive fiber sensors can detect the local deformations of the sweater caused by a sitting posture change, and the collected signals are then transmitted to an Arduino microcontroller for further processing and analysis in order to evaluate the postures; finally, audible and light feedback will be given for personal prevention. As shown in Figure 8e,f and the corresponding video (Supplementary Materials, Videos S1 and S2), poor body postures that cause an increase in sensor resistance, including excessive head forwards and kyphosis (yellow part), were successfully detected by the system, and then a buzzer alarm was activated to alert the wearer.

## 4. Conclusions

In this study, MXene/WPU composite-coated fiber strain sensors with favorable sensing performance were successfully fabricated by a facile and quick method, i.e., simply dip-coating elastic PU fibers into homogeneous MXene/WPU dispersion. Electrostatic repulsion between negatively charged MXene and WPU plays a critical role in the uniform dispersion of MXene nanosheets in a WPU solution. Moreover, due to the abundant surface functional groups in MXene and WPU, together with the identical PU polymer constituent, very firm bonding was achieved between an MXene/WPU conductive polymer composite and stretchable PU matrix; meanwhile, very small Young’s modulus mismatch existed between them, all of which endow the fiber strain sensors with the super resistant capability to washing, peeling, and cyclic stretching–releasing. Moreover, thanks to the high sensitivity and large strain working range, we investigated the applicability of such fiber strain sensors in human motion monitoring from vigorous joint movements and expression change to subtle wrist pulses. Finally, a smart data glove for finger bending angle and hand gesture monitoring and a prototype system for human body posture monitoring and correction were achieved by embedding the fiber strain sensors into ordinary fabrics, fully demonstrating the great potential of MXene/WPU composite-coated fiber strain sensors that are wearable for motion and healthcare monitoring.

## Data Availability

The data that support the findings of this study are available from the corresponding author upon reasonable request.

## Supplementary Materials

The following supporting information can be downloaded at https://www.mdpi.com/article/10.3390/s24010271/s1, Figure S1. SEM images of (a) Ti_3_AlC_2_, (b) multi-layer Ti_3_C_2_T_x_, and (c) few-layer Ti_3_C_2_T_x_ nanosheets. (d) XRD patterns of Ti_3_AlC_2_ and Ti_3_C_2_T_x_. Figure S2. Solution photos after mixing MXene dispersion with (a) negatively charged WPU and (b) ordinary WPU. Video S1: Smart clothing for monitoring kyphosis. Video S2: Smart clothing for monitoring head forward.
