# Supplementary material for: A Flexible and Stretchable MXene/Waterborne Polyurethane Composite-Coated Fiber Strain Sensor for Wearable Motion and Healthcare Monitoring
Source: Sensors (Basel). 2024 Jan 2;24(1):271. doi: 10.3390/s24010271 (PMC10781211; doi:10.3390/s24010271)
Supplement: Supplementary file 1 [file sensors-24-00271-s001.zip › supplementary materials.zip!/supplementary materials.pdf]

Supplementary Materials:

A Flexible and Stretchable MXene/Waterborne Polyurethane Composite-Coated Fiber Strain Sensor for Wearable Motion and Healthcare Monitoring

*Junming Cao[1,2], Yuanqing Jiang[3], Xiaoming Li[3], Xueguang Yuan[1,2], Jinnan Zhang[1,2], Qi He[1,2], Fei Ye[1,2], Geng Luo[1,2], Shaohua Guo[1,2], Yangan Zhang[1,2], and Qi Wang[1,2,]**

1 State Key Laboratory of Information Photonics and Optical Communications, Beijing University of Posts and Telecommunications, Beijing, 100876, People's Republic of China

2 School of Electronic Engineering, Beijing University of Posts and Telecommunications, Beijing, 100876, Peo-ple's Republic of China

3 No.208 Research Institute of China Ordnance Industries, Beijing, 102202, People's Republic of China

* Correspondence: wangqi@bupt.edu.cn

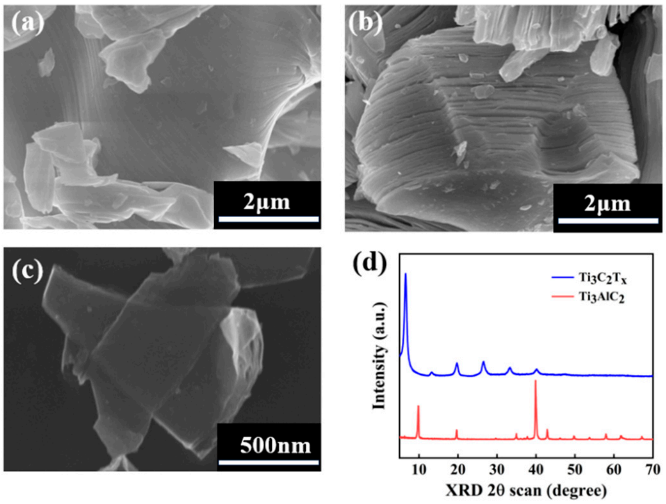

Figure S1. SEM images of (a) Ti_3AlC_2, (b) multi-layer $Ti_3C_2T_x$, and (c) few-layer $Ti_3C_2T_x$ nanosheets. (d) XRD patterns of Ti_3AlC_2 and $Ti_3C_2T_x$

SEM images of Ti3AlC2, multi-layer $Ti_3C_2T_x$, and few-layer Ti_3AlC_2 are shown in Figures S1 (a-c), respectively. Ti_3AlC_2 raw material had typical structure of layered MAX phase (Figure S1a). After etching out Al atomic layers in Ti_3AlC_2 precursors, an accordion-like multi-layered structure was formed in the resulting $Ti_3C_2T_x$ (Figure S1b). Followed by centrifugal separation, $Ti_3C_2T_x$ nanosheets with few layer was obtained. Successful removal of Al atomic layer by chemical etching and the formation of Ti_3C_2Tx MXene can be further demonstrated by XRD pattern evolution (Figure S1d), in which the most intense (104) peak originally locating near 40.7° in the XRD pattern of Ti_3AlC_2 MAX precursor disappeared and another strong (002) peak was shifted from 10.2° in Ti_3AlC_2 MAX to 7.5° in Ti_3AlC_2 MXene due to the increased spacing between lamellae.

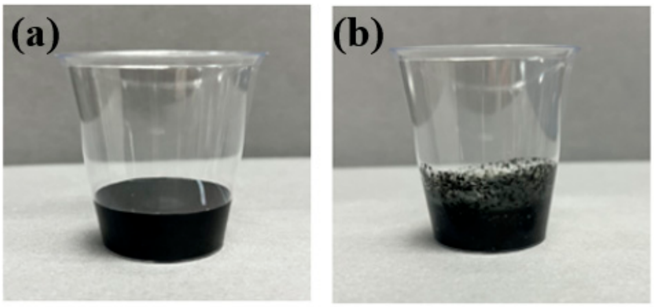

Figure S2. Solution photos after mixing MXene dispersion with (a) negatively-charged WPU, and (b) ordinary WPU

The photo of the solution formed by mixing negatively-charged WPU and MXene was shown in Figure S2(a) and noticeable agglomeration or sedimentation could not be observed, demonstrating very good dispersion of MXene nanosheets. While in the comparison group, blending MXene with ordinary WPU induced significant agglomeration and sedimentation, as shown in Figure S2(b).
